# Reduced phloem uptake of *Myzus persicae* on an aphid resistant pepper accession

**DOI:** 10.1186/s12870-018-1340-3

**Published:** 2018-06-27

**Authors:** Mengjing Sun, Roeland E. Voorrips, Greet Steenhuis-Broers, Wendy van’t Westende, Ben Vosman

**Affiliations:** Plant Breeding, Wageningen University & Research, P.O. Box 386, 6700 AJ Wageningen, The Netherlands

**Keywords:** Aphid resistance screening, *Capsicum baccatum*, EPG, Callose, Real-time PCR

## Abstract

**Background:**

The green peach aphid (GPA), *Myzus persicae*, is economically one of the most threatening pests in pepper cultivation, which not only causes direct damage but also transmits many viruses. Breeding aphid resistant pepper varieties is a promising and environmentally friendly method to control aphid populations in the field and in the greenhouse. Until now, no strong sources of resistance against the GPA have been identified. Therefore the main aims of this study were to identify pepper materials with a good level of resistance to GPA and to elucidate possible resistance mechanisms.

**Results:**

We screened 74 pepper accessions from different geographical areas for resistance to *M. persicae*. After four rounds of evaluation we identified one *Capsicum baccatum* accession (PB2013071) as highly resistant to *M. persicae*, while the accessions PB2013062 and PB2012022 showed intermediate resistance. The resistance of PB2013071 resulted in a severely reduced uptake of phloem compared to the susceptible accession, as determined by Electrical Penetration Graph (EPG) studies. Feeding of *M. persicae* induced the expression of callose synthase genes and resulted in callose deposition in the sieve elements in resistant, but not in susceptible plants.

**Conclusions:**

Three aphid resistant pepper accessions were identified, which will be important for breeding aphid resistant pepper varieties in the future. The most resistant accession PB2013071 showed phloem-based resistance against aphid infestation.

**Electronic supplementary material:**

The online version of this article (10.1186/s12870-018-1340-3) contains supplementary material, which is available to authorized users.

## Background

Pepper (*Capsicum* spp.) belongs to the *Solanaceae* family and is one of the economically most important and widely cultivated vegetable crops. The annual global production area and yield of pepper are 3.7 million hectares and 37 million tons, respectively (FAOSTAT, 2015). The genus *Capsicum* originates from Central and South America and 25 distinct species have been reported [1], among which five are domesticated: *C. annuum*, *C. chinense*, *C. frutescens*, *C. baccatum*, and *C. pubescens* [2].

Aphids (*Aphididae*) are the most wide-spread pest insects. More than 100 aphid species are reported as economically important pests and most crops suffer from one or more species [3]. The green peach aphid (GPA), *Myzus persicae*, is one of the most threatening pests in pepper and many other crops. It is a generalist that causes many types of damages in pepper, including chlorosis, necrosis, wilting, defoliation and flower and fruit abortion. It produces honeydew when feeding on plants, which may affect fruit quality and reduce photosynthetic capacity by stimulating mold development. However the most serious damage is done indirectly by the viruses that GPA may vector, including Potato virus Y, Pepper mottle virus, Pepper severe mosaic virus, Pepper yellow mosaic virus, and Peru tomato mosaic virus [4].

As phloem-feeding insects, aphids use their specialized mouthparts, the stylets, to penetrate plant tissue and to take up nutrients without inflicting serious damage [5, 6]. To study aphid probing and feeding behaviour, the electrical penetration graph (EPG) technique can be used [7]. In the EPG technique an aphid and a plant are wired into an electrical circuit, and aphid activity on the plant is recorded as waveforms that are specific for different probing and feeding activities [8, 9]. The EPG technique can be applied to explore the nature of the differences in aphid behaviour on resistant and susceptible plants, for instance to determine where in the leaf an aphid encounters a specific plant resistance factor [5, 10–12].

In several cases it has been observed that aphids show a significantly shorter period of phloem feeding on resistant than on susceptible plants [11, 12]. One possible explanation is occlusion of the phloem vessels in response to feeding [13, 14], which may be caused by callose deposition [13, 15]. Callose, a ß-1,3-glucan, is an important component in the defense response to mechanical wounding, pathogen infection and insect infestation [16–18]. In *Arabidopsis thaliana* callose deposition was induced and the expression of related synthase genes was enhanced in response to whitefly infestation [19]. In rice, callose deposition was suggested as an important resistance factor against the brown plant hopper [15].

Callose is produced by callose synthases (CalS), which are encoded by a family of callose synthase genes. Twelve, ten, six, nine and eight synthase genes were identified and characterized in *A. thaliana* [20, 21], rice [22], barley [23], wheat [24] and grapevine [25], respectively. These genes were studied in detail in *A. thaliana*. The *CalS7* gene was reported to be expressed specifically in the phloem vessels and was responsible for callose deposition induced by mechanical wounding [26]. The *CalS12* was mainly shown to be required for wound and papillary callose formation in response to pathogen attack [27, 28] and to aphid feeding [29]. The expression of *CalS1* was found to be up-regulated after infestation with aphids and whiteflies [19, 30]. Besides the role of callose formation and deposition in plant resistance, the breakdown of callose might be another factor. Callose degradation, which is governed by some ß-1,3-glucanases, was shown to cause susceptibility in the interaction between the brown plant hopper and rice [15] as well as in the interaction between bird cherry-oat aphid and barley [31].

Due to the severe negative effects of aphids on crop yield and quality, chemical pesticides have been widely used to control aphids. However, with more and more reports on aphids developing resistance to pesticides [32, 33] and growing concern about the environmental impact of insecticides, breeding aphid resistant pepper varieties is a desirable alternative which will become an indispensable part of integrated pest management. Plant resistance mechanisms against insects, including aphids, are classified as antixenosis, antibiosis and tolerance [34–37]. Antixenosis, or non-preference, affects insect settling or feeding through repellence or deterrence [38]. Antibiosis-based resistance impairs insect survival, growth, development and fecundity, caused by chemical or morphological adaptations of the plant [36, 39, 40]. Tolerance reduces damage to the plant after insect feeding, in spite of the presence of insect population densities similar to those on susceptible plants [34, 40]. A number of genes conferring resistance to aphids have been identified in crops, including among others in wheat [41], soybean [42], lettuce [43] and cowpea [44]. However, only two genes have been cloned, the tomato *Mi-1.2* gene which confers resistance to the potato aphid *Macrosiphum euphorbiae*, to the whitefly *Bemisia tabaci* and to three species of root-knot nematodes [45–47], and the melon *Vat* gene, which confers resistance to the cotton aphid *Aphis gossypii*, as well as to non-persistent viruses when vectored by *A. gossypii* [48]. Both genes are of the NBS-LRR type [45, 48] and work according to the gene-for-gene principle which means that the *R* gene in the plant recognizes an effector secreted by the aphid, and activates an aphid-specific defense response [35]. Until now only a few studies to identify donors of resistance genes that may be used in pepper breeding have been published [49, 50]. One *C. pubescens* plant showed antixenosis rather than antibiosis resistance to the GPA [49], but detailed information on this accession was not provided, and no hybridization between *C. pubescens* and *C. annuum* has been reported yet. Franz *et al.* detected significant differences among 50 pepper accessions in choice tests with GPA, however no strong resistance was found [50]. De Costa *et al*. identified a pepper cultivar which was resistant against the *A. gossypii*, but it is unknown if it is also resistant to GPA [51]. Therefore, there is still an urgent need for pepper accessions resistant to GPA.

This research was carried out to identify accessions with a good level of resistance to GPA and to shed light on the possible resistance mechanism. We evaluated a collection of *C. annuum*, *C. chinense*, *C. frutescens* and *C. baccatum* accessions for GPA resistance and identified resistant accessions in *C. baccatum.* The resistance, mainly affecting aphid reproduction, is most likely phloem based and accompanied by callose deposition.

## Results

### Selection of pepper accessions resistant to GPA

Evaluation of 50 accessions, representing 4 *Capsicum* species, for GPA resistance showed large and highly significant differences (Additional file 1: Table S1) for the two resistance parameters used: survival of the original nymphs and the number of next generation nymphs produced. Survival rate ranged from 6 to 97%, while the average number of new nymphs produced by each living adult during infestation varied from 0 to 0.8.

**Fig. 1:**
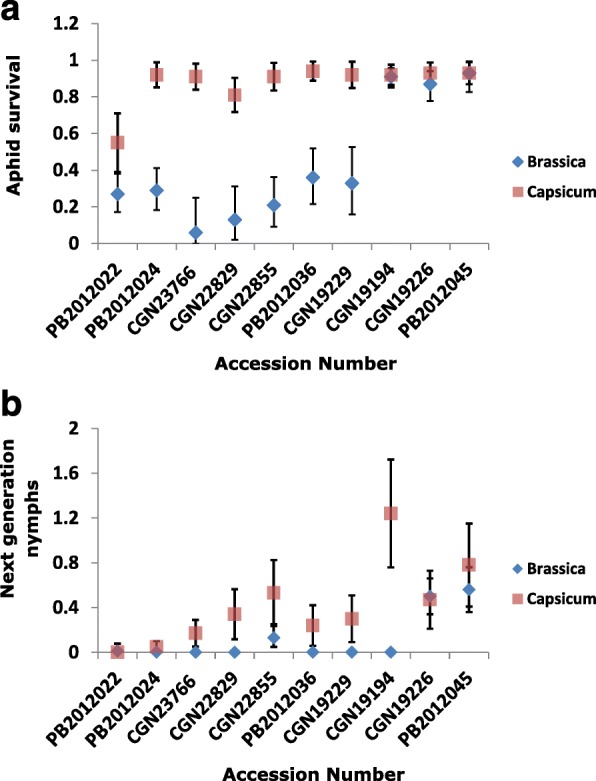
Performance of *M. persicae* after adaptation to different host plants. Ten selected accessions were infested with aphids reared on Chinese cabbage (*Brassica rapa* cv. Granaat; blue icon) or pepper (*C. annuum* CGN19226; red icon). Performance parameters used: survival of the original nymphs (**a**) and the number of next generation nymphs produced (**b**). Survival was determined by dividing the number of living aphids by the total number of aphids (dead and alive) in the clip cage. The number of next generation nymphs was divided by the average number of living aphids present, calculated as (2*living aphids + dead aphids)/2. Each bar represents the mean values ± SD. More details on the statistics can be found in Additional file 1: Table S1

After transferring the GPA rearing from Chinese cabbage to *C. annuum* accession CGN19226, ten selected accessions (Additional file 1: Table S1 and Fig. 1) were re-tested with the GPA colony that had been adapted to pepper. These included seven accessions showing a low aphid survival and also a low production of second generation nymphs in the first experiment. The accessions *C. annuum* CGN19226 and *C. frutescens* PB2012045 were chosen as susceptible standards as they are from different species and origins. Accession *C. annuum* CGN19194 was selected as no second generation nymphs were produced on it, while the number of surviving adults was high, suggesting that this accession may possess a resistance mechanism affecting reproduction only. In this second experiment the two susceptible standards were again completely susceptible. Accession CGN19194 was also highly susceptible; the reduced reproduction observed in the first test was not confirmed in the second one using the aphids adapted to pepper. Among the seven accessions selected as resistant in the first experiment, the five *C. chinense* accessions respectively showed varying levels of resistance based on the two resistance parameters between the two experiments (T-test, *P* < 0.01). However, the two *C. baccatum* accessions (PB2012022 and PB2012024) continued to show an impaired reproduction in the second experiment, which was the same as that in the first experiment.

**Fig. 2:**
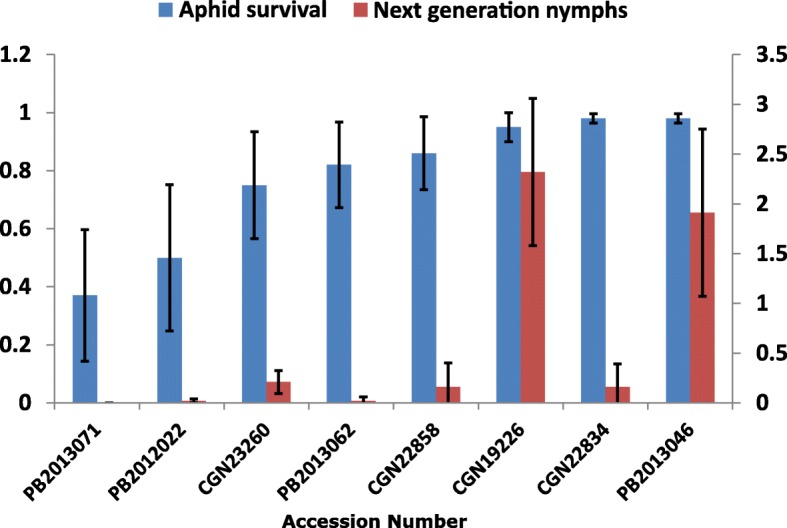
Performance of *M. persicae* on eight accessions after adaptation on *C. baccatum*. Aphids were reared on accession PB2013046. Performance parameters used: survival of the original nymphs (blue column) and the number of next generation nymphs produced (red column). Survival was determined by dividing the number of living aphids by the total number of aphids (dead and alive) in the clip cage. The number of next generation nymphs was divided by the average number of living aphids present, calculated as (2* living aphids + dead aphids)/2. Each bar represents the mean values ± SD. More details on the statistics can be found in Additional file 2: Table S2

Based on the results of the initial screening, we decided to focus further efforts on the screening of *C. baccatum* accessions (Additional file 2: Table S2). In the third experiment accession *C. annuum* CGN19226 was used as susceptible standard. Evaluation of 38 accessions showed significant variation for aphid survival and aphid fecundity: survival of original nymphs varied from 0.49 to 0.98 and the number of new nymphs produced per aphid ranged from 0 to 0.89. The accessions PB2013071, PB2013062 and CGN23260 were among the most resistant although they were not significantly different from a number of others, based on aphid survival and next generation nymphs produced. Accession PB2012022 showed a slightly higher nymph survival, but no next generation nymphs, confirming previous results. The accession *C. baccatum* PB2013046 was as susceptible as the susceptible standard *C. annuum* accession CGN19226. For this reason we transferred the GPA rearing to PB2013046 and re-tested eight accessions for resistance using GPA reared on this susceptible *C. baccatum* accession (Additional file 2: Table S2 and Fig. 2). In this fourth experiment, we classified PB2013071, PB2013062, CGN23260 (no reproduction, relatively low survival: < 0.7) together with PB2012022 and CGN22834 (also no reproduction, somewhat higher survival: > 0.7) as resistant, CGN22858 (some reproduction, low survival) as an intermediate resistant, and PB2013046 together with CGN19226 (high reproduction, high survival) as susceptible accessions. In this experiment, the accession *C. baccatum* PB2013071 was again the most resistant, as it continued showing the lowest survival and no reproduction. The accession *C. baccatum* PB2013046 was again as susceptible as *C. annuum* accession CGN19226. The correlation coefficient between the number of new nymphs produced by *C. annuum* and *C. baccatum* adapted aphids (third and fourth experiment) was 0.83, which was calculated on the basis of the eight accessions tested with both populations.

### GPA population development on selected accessions

The three selected resistant *C. baccatum* accessions (PB2013071, PB2013062 and PB2012022), the susceptible *C. baccatum* accession (PB2013046) and the susceptible *C. annuum* accession (CGN19226) were used for further confirmation of resistance and susceptibility using a population development experiment. Results are shown in Table 1. PB2013046 is confirmed as a susceptible accession on which aphids show a high survival rate and strong fecundity, which was even higher than the *C. annuum* susceptible standard (CGN19226). Accession PB2013071 showed the highest level of resistance, while the accessions PB2013062 and PB2012022 were intermediate.

**Table 1:** Population development of the aphid *M. persicae* on five *Capsicum* accessions

Accession number	Adults		Nymphs^a^
PB2013071	33	a	0.7
PB2012022	158	b	1.2
PB2013062	337	c	1.6
PB2013046	2655	d	2.0
CGN19226	1633	d	2.0

^a^Average number of nymphs according to visual scale: 0 = none, 1 = few (< 50), 2 = many (> 50)

Mean values of adult count followed by the same letter are not significantly different (LSD- test on log-transformed scale at *P* < 0.05)

### EPG analysis on accessions PB2013071 and PB2013046

Results for the parameters extracted from the EPG recordings are presented in Table 2. No significant difference was found between the resistant accession PB2013071 and the susceptible accession PB2013046 for parameters related with non-probing, pathway phase, derailed stylet mechanics and xylem phase. However, significant differences were seen during the phloem phase E1 (salivation into the phloem) and E2 (phloem sap ingestion) (T-test, *P* < 0.05). The total duration of E1 on PB2013071 was more than two times as long as on PB2013046, while the total duration of E2 on PB2013071 was only about one-eighth of that on PB2013046. However, there was no significant difference in the number of aphids that successfully reached phloem ingestion E2: 75% on PB2013046 and 47% on PB2013071 (Fisher exact test, *P* = 0.101). The total number of individual cell punctures (potential drops) and average number of potential drops per minute of pathway phase were both more on PB2013071 than PB2013046 (T-test, *P* < 0.01).

**Table 2:** *M. persicae* EPG parameters measured on a susceptible (PB2013046) and a resistant (PB2013071) *C. baccatum* accession

Class	Trait Definition^a^	2,013,046	2,013,071	*P*-value
Non-probing (NP)	Number of NP	15.7	17.3	0.5645
	Total duration of NP (min)	21.3	21.3	0.9879
Probes	Number of Probes	14.8	16.3	0.5870
	Total duration of Probes (min)	338.6	338.6	0.9874
Pathway phase (C)	Number of C (pathway periods)	24.4	27.8	0.3528
	Total duration of pathway period (min)	125.0	155.1	0.1206
Derailed stylet (F)	Number of periods with F form	3.3	1.5	0.0650
	Total duration of F period (min)	72.9	59.8	0.6230
Xylem phase (G)	Number of periods with G form	2.1	1.7	0.5651
	Total duration of G period (min)	32.9	33.4	0.9686
	Time to first G phase (min)	162.8	153.2	0.8326
Phloem phase (E)	Number of salivation periods (E1)	6.1	9.4	0.0265
	Time to first E1 (min)	105.1	82.2	0.4575
	Total duration of E1 (min)	28.6	80.0	0.0001
	Total duration of phloem uptake (E2, min)	78.9	10.5	0.0032
	Time to first E2 (min)	258.5	324.0	0.0414
	Number of E1 followed by E2	1.2	0.3	0.0049
	Total duration of E1E2 (min)	86.6	5.5	0.0008
	Time to first E1E2 (min)	225.4	324.4	0.0037
Potential drops (Pd)	Number of potential drops	83.7	156.4	0.0000
	Number of Pd per min of Pathway C	0.7	1.0	0.0025
Aphids reaching E2	Percentage of aphids reaching E2	75%	47%	0.1010

Data are based on 20 and 17 aphids tested on PB2013046 and PB2013071, respectively. Mean values are shown

### Callose deposition

**Fig. 3:**
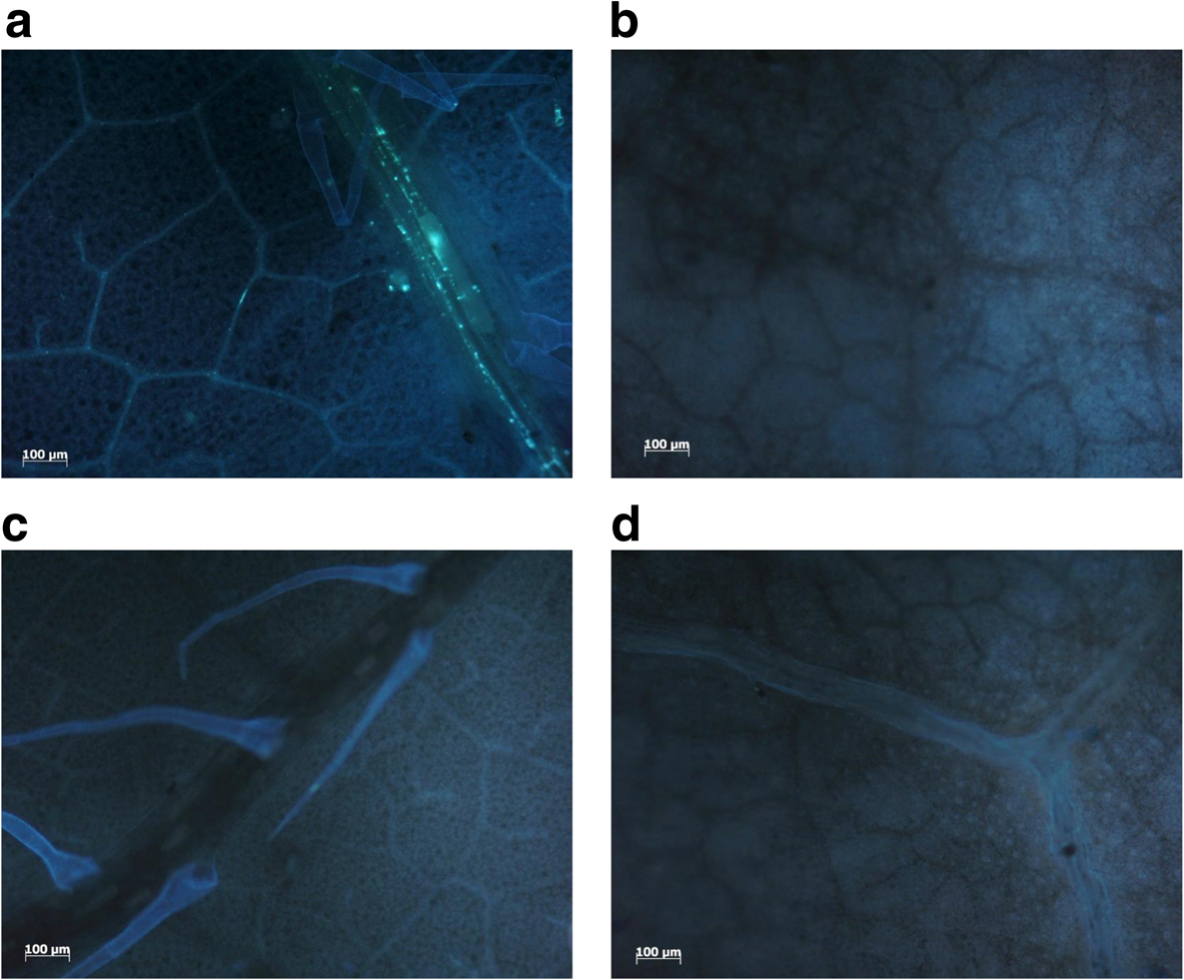
Histochemical staining of callose in the GPA-infested leaves (**a**, **b**) and GPA-free leaves **(c, d).** Resistant accession PB2013071 (**a**, **c**); susceptible accession PB2013046 (**b**, **d**). Staining was carried out 24 h after the start of the infestation

Callose deposition is considered important for plant resistance against pathogens and insects [15, 52]. We studied the accumulation of callose in resistant and susceptible plants after GPA feeding. Detached leaves were infested with GPA for 24 h, after which three or four leaf disks were prepared for the callose deposition study. Representative results are shown in Fig. 3 and additional images can be found in Additional file 3: Figure S1. Callose signals were detected in the vascular tissue of all sampled leaf disks from accession PB2013071, but not in accession PB2013046 treated by GPA or in leaf disks of both accessions without aphids infestation.

### Identification and expression of callose related genes

Nine putative *Callose Synthase* (*CalS)* genes were identified in the *C. annuum* sequences and named with reference to the most homologous gene in *Arabidopsis*, *CaCalS1*, *CaCalS3*, *CaCalS5*, *CaCalS7*, *CaCalS8*, *CaCalS9*, *CaCalS10*, *CaCalS11* and *CaCalS12*. The length of open reading frames (ORFs) and gene IDs in both pepper genome sequences are listed in Additional file 4: Table S3. A neighbour-joining tree of CalS proteins among pepper, *Arabidopsis* and grapevine is shown in Additional file 5: Figure S2.

**Fig. 4:**
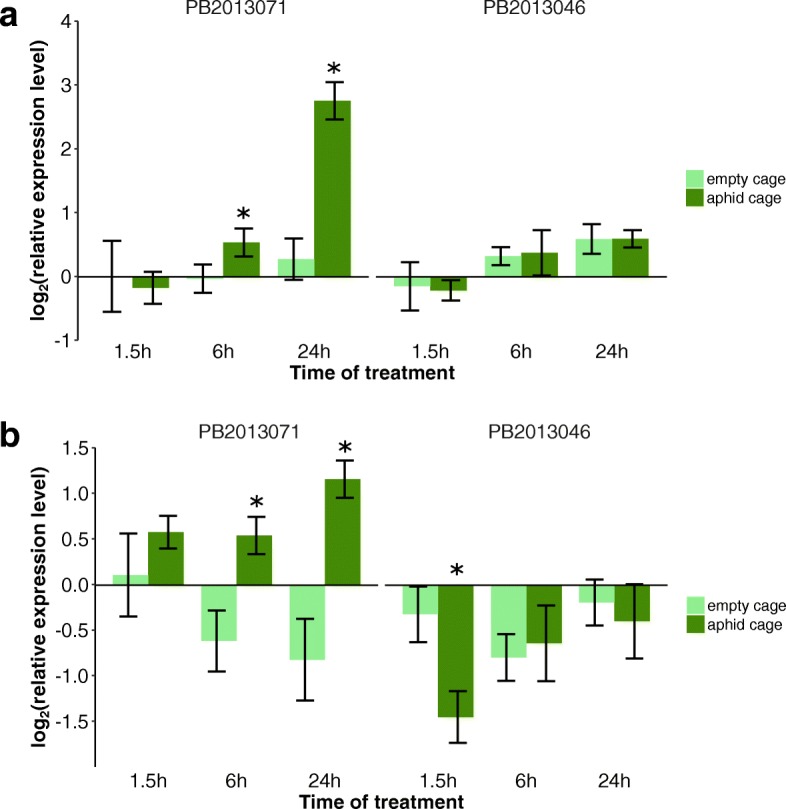
Expression analysis of *Callose synthase* genes *CalS1* (**a**) and *CalS7* (**b**) after aphid infestation. Gene expression was quantified relative to the value obtained from leaf samples without clip cage or aphid infestation (time point 0 h). Data was log2-transformed. Each bar represents the mean values of three or four biological replicates, each with two technical replicates. The *actin* gene was used as the reference gene. * indicates a significant difference in level of gene expression between the GPA treated sample and the GPA-free (empty clip cage) sample at that time points (T-test, *P* < 0.05). Each bar represents the mean values ± SD

To shed light on the regulation of the callose deposition we compared the expression of *callose synthase* genes (*CalS* family genes) and the *basic ß-1,3-glucanase* gene (*BGLU*) in GPA-infested leaves with those of non-infested leaves. Nine putative *CalS* family genes were analyzed by real-time PCR. Among these nine genes, only *CalS1* (Fig. 4a) and *CalS7* (Fig. 4b) showed a clear change in transcript accumulation upon aphid infestation. In the leaves of PB2013071 infested with GPA, no difference in expression was detected for both genes after 1.5 h, but expression was significantly up-regulated at 6 h and 24 h after the start of the infestation compared to empty cages (T-test, *P* < 0.05). The expression level of *CalS1* increased 5.6-fold (T-test, *P* = 0.0004) and that of *CalS7* increased 3.9-fold (T-test, *P* = 0.0088) 24 h post-infestation compared to empty cages. In the leaves of PB2013046 infested with GPA, expression of the *CalS1* and *CalS7* genes remained stable during 24 h, except that *CalS7* after 1.5 h showed significantly lower expression level in GPA infested leaves compared to GPA free leaves (T-test, *P* = 0.0017). The expression of the *CalS1* and *CalS7* gene in leaves of both accessions after 1.5 h, 6 h, 24 h with empty clip cages remained constant (ANOVA, *P* > 0.05).

**Fig. 5:**
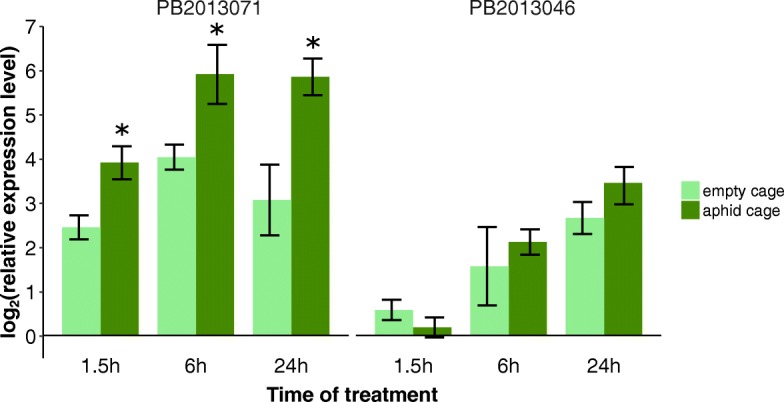
Expression analysis of the *BGLU* gene after aphid infestation. Gene expression was quantified relative to the value obtained from leaf samples without clip cage or aphid infestation (time point 0 h). Data was log2-transformed. Each bar represents the mean values of three or four biological replicates, each with two technical replicates. The *actin* gene was used as the reference gene. * indicates a significant difference in level of gene expression between the GPA treated sample and the GPA-free (empty clip cage) sample at that time points (T-test, *P* < 0.05). Each bar represents the mean values ± SD

The *BGLU* gene was up-regulated in PB2013071 at all three time-points during the 24 h of aphid infestation compared to empty cages (Fig. 5) (T-test, *P* < 0.05). The ratio of transcripts with and without aphid infestation increased to 2.3 at 1.5 h (T-test, *P* = 0.0070), to 3.9 at 6 h (T-test, *P* = 0.0062) and to 6.4 at 24 h (T-test, *P* = 0.0141) after the start of the infestation. In contrast, there was no significant difference in expression of the *BGLU* gene in PB2013046 between plants with GPA treatment for 1.5 h, 6 h and 24 h and plants with empty cages at the same time points. In leaves that received empty clip cages, the expression of the *BGLU* gene increased after 1.5 h, 6 h, 24 h, in both accessions (ANOVA, *P* < 0.05).

## Discussion

### Importance of rearing history during evaluation of GPA performance

When the initial evaluations were performed using GPA reared on cabbage or pepper, large differences were seen in aphid survival: GPA survival was relatively low when cabbage reared GPA were used and high when pepper reared GPA were used. The effect of GPA rearing history varied among *Capsicum* accessions. There was hardly any effect on *C. annuum* accessions, whereas on *C. chinense* accessions the effect of the rearing was pronounced. It has been reported before that the host plant on which an aphid colony is reared can affect the performance of aphids. For example, the grain aphid *Sitobion avenae* reared on wheat performed less well on the cocksfoot than on wheat [53], and *A. gossypii* that adapted to cotton or cucumber could not survive and reproduce after reciprocal host transfer [54].

About the background of host adaptation in our test system we can only speculate. (1) As there are differences in metabolite content between cabbage and pepper, aphids may have to develop/adjust their detoxification system to adapt to the host plant, which may take several generations. For instance, the enzymatic detoxification system, a family of glutathione S-transferases, was reported to be involved in adaptation of GPA to different species containing different glucosinolates [55]. (2) Another hypothesis involves a change in endosymbiont composition after transferring from one plant species to the other. Mutualistic symbionts play an instrumental role in plant-insect interactions [56]. Host plant specialization of pea aphid *Acyrthosiphon pisum* was reported to be influenced by the facultative pea aphid U-type symbiont (PAUS) [57]. Also, the abundance of *Buchnera aphidicola*, the primary endosymbiotic bacterium of GPA, was found to affect GPA host acceptance and stylet penetration on host plants [58]. In our case, the rearing on *C. annuum* may have changed the aphid metabolism, introduced a new endosymbiont species or increased the abundance of an already present symbiont species, improving their performance on *C. chinense*. Based on the observations made, it is highly recommended that evaluations of germplasm are carried out using insect populations that are adapted to the species, or that re-testing is conducted with adapted aphids to confirm results of resistance screenings especially when aphids are reared on evolutionary distant plant materials, as on Chinese cabbage in our case.

### A wide diversity in GPA resistance among *Capsicum* accessions

The high multiplication rate of aphids makes them a pest in many crops [59]. Even in the presence of natural enemies (predators and parasitoids) it is often difficult to control the growth of aphid populations. Varieties that are highly or even partly resistant to aphids can make a big difference by reducing the multiplication rate of the aphids and thus give natural enemies more chance to control them [60]. To develop such varieties, resistance sources need to be identified in crossable species and in this paper we describe the identification of such sources. Accessions from four inter-crossable *Capsicum* species were evaluated for resistance against the GPA and considerable variation was observed. After four rounds of evaluation, we identified a number of *C. baccatum* accessions with a relatively high and stable level of aphid resistance. A GPA population development experiment among five selected accessions confirmed their resistance. Resistance primarily seems to affect the production of next generation nymphs and to a lesser extent the survival of the aphid itself. Accession *C. baccatum* PB2013046 showed susceptibility with the highest GPA survival rate and fecundity while *C. baccatum* PB2013071 showed the strongest resistance, with a significantly lower GPA survival than on the susceptible accession and a severely impaired population development. Accessions *C. baccatum* PB2013062 and *C. baccatum* PB2012022 showed intermediate levels of resistance. These three accessions are the first *C. baccatum* accessions in which resistance to GPA is demonstrated and may be used for breeding resistant varieties in the other *Capsicum* species as well. The species *C. baccatum* has been used for pepper breeding as donor of anthracnose [61, 62] and powdery mildew resistance [63]. With respect to insect resistance, two *C. baccatum* accessions were reported as a good source for thrips (*Thrips parvispinus* and *Frankliniella occidentalis*) resistance [64] and three *C. baccatum* accessions were identified as tolerant but not resistant to cotton aphid (*A. gossypii*) [50]. To our knowledge, this is the first report of a strong antibiosis type of resistance to GPA in *Capsicum*.

### Impaired phloem uptake on a resistant accession

The Electrical Penetration Graph (EPG) technique allows an in-depth study of the feeding behaviour of piercing-sucking insects [7] and is able to reveal possible constraint encountered by such insects when trying to feed on plants [5, 9]. The EPG analysis revealed significant differences in parameters related with the phloem phase of GPA feeding on the resistant versus susceptible pepper accession. In comparison to the susceptible accession PB2013046, on the resistant PB2013071 the phloem salivation periods were longer and more frequent, and the phloem uptake periods were much shorter, suggesting that the resistance is most likely located in the phloem. In other words, aphids feeding on resistant accession PB2013071 have difficulties to initiate and sustain phloem sap ingestion. Aphids feeding on accessions containing a phloem based resistance are likely to grow more slowly, have lower fecundity and are more likely to die early due to the problems they experience with taking up sufficient nutrition. This is in line with our observations. Besides the possibility to control aphid population, phloem based resistance may reduce the transmission of persistent viruses because generally aphids cannot acquire persistent viruses during short-time feeding [65]. It is likely that the percentage of plants infested with persistent viruses will also decrease when the number of aphids carrying virus is low [66].

No significant differences were observed in the pre-phloem phase, with the exception of the number of potential drops. Potential drops indicate that the aphid’s stylets puncture cells along the pathway to the phloem [67]. The number of potential drops was much higher on the resistant accession PB2013071 than on the susceptible accession PB2013046. One biotype of soybean aphid (*Glycine max*) was also shown to have a higher number of potential drops when feeding on resistant genotypes than on susceptible genotypes [11]. It has been reported that potential drops are related with aphid transmission of non-persistently transmitted viruses [68, 69]. However, it is unknown if they are indicative for a specific plant resistance component. In spite of the difference in number of potential drops, the total duration of the pathway phase was not different between the two accessions. We examined the number of cell layers between the epidermis and the phloem in the two accessions, which might have a relation with the number of cells punctured while passing to the phloem; however we did not observe a difference between the two accessions in this respect (results not shown). Therefore, it remains unclear if the higher number of potential drops on the resistant plant is important for resistance.

### Induced callose deposition in the resistant accession

One possible mechanism of phloem-based resistance might be occlusion of the phloem vessels in response to aphid feeding, which may result from callose deposition. Callose induction and formation is a defense response to phloem-sucking pests that plugs the sieve element to obstruct feeding [15, 30, 70–72]. Our data clearly show callose deposition 24 h after the start of the aphid infestation on detached leaves from the resistant accession PB2013071, but not on the susceptible accession PB2013046 and also not on non-infested leaves of either accession. This suggests that callose deposition may be one of the mechanisms behind the phloem-based resistance. The fact that callose deposition was studied on detached leaves and not on intact plants may have resulted in a weaker callose response. We did not assess the resistance on detached leaves, but studies on lettuce with the aphid *Nasonovia ribisnigri* [73] suggest that the expression of resistance may be partially reduced in detached leaves compared to intact plants. It is also reported that callose deposition is observed in epidermal and mesophyll cell walls in the interaction of *A. gossypii* with melon plants carrying resistance gene *Vat* [74].

As a strong callose signal was found in leaf veins of resistant pepper plants after GPA feeding and not in susceptible plants, it was hypothesized that one or several *CalS* family genes or *ß-1,3-glucanase* gene(s) might be involved in this difference between resistant and susceptible plants after GPA infestation. We carried out quantitative real-time PCR to examine whether callose deposition could be due to increased *CalS* gene expression upon aphid attack. Among the nine putative *CalS* family genes, the *CalS1* gene was found to be significantly up-regulated at 6 h and 24 h post-infestation of GPA feeding in the leaves of PB2013071, while the level of gene transcripts remained constant in the leaves of PB2013046 during the initial 24 h of aphid infestation. The *CalS1* gene has been reported in *Arabidopsis* to accumulate after whitefly and aphid infestation [19, 30]. Besides the *CalS1* gene, we detected that transcripts of *CalS7* in the infested leaves of resistant accession PB2013071 also significantly increased after 6 h and 24 h compared to non-infested leaves, but less than the transcripts of *CalS1*. The *CalS7* gene is the only phloem-specific *callose synthase* gene and it is responsible for callose biosynthesis in developing sieve elements as well as for callose deposition after mechanical wounding in mature phloem [26]. Here we report for the first time an induction of *CalS7* transcription upon infestation with a phloem-feeding insect. The expression of the two *CalS* genes increased after aphid attack in leaves of the resistant accession but not in leaves of the susceptible accession. We speculate that the *CalS1* and/or *CalS7* genes are responsible for callose deposition in leaves of the resistant accession PB2013071 after GPA feeding. As in *A. thaliana CalS1* also can be induced by other phloem-feeding insects like the whitefly *B. tabaci* [19] and cabbage aphid *Brevicoryne brassicae* [30], the *CalS1* gene might have a common role in callose deposition induced by phloem-feeders. As the *CalS7* gene is expressed specifically in phloem vessels [75], the sampling of entire leaf disks rather than just leaf veins for real-time PCR may lead to an underestimation of the level of induction in phloem punctured by the insect. The role of these two *CalS* genes in callose deposition needs to be further studied. As transformation of pepper is difficult [76], it may not be so easy to do this by silencing the two *CalS* genes. It may be more effective to carry out a genetic (fine) mapping study to identify genes involved in the resistance.

The BGLU protein, also known as pathogenesis-related (PR) protein 2, is responsible for hydrolyzing callose (ß-1,3-glucan) in order to destabilize the cell wall of pathogens as well as to activate some immunity elicitors which can stimulate defense responses against pathogen attack [77]. In pepper plants BGLU has been reported to play an important role during defense against pathogens [78–80]. The BGLU protein or *BGLU* gene transcript has also been found to accumulate in leaves of wheat [81] and *Arabidopsis* [82] after aphid infestation. The *BGLU* gene is considered as a marker of the salicylic acid (SA)-dependent defense response in plants [83, 84]. Also, some ß-1,3-glucanases of the same family as BGLU were proposed as susceptibility factors in the interaction between brown plant hopper and rice [15] as well as between bird cherry-oat aphid and barley [31]. It is thought that the feeding barrier for insects caused by callose deposition can be weakened in susceptible plants due to accumulation of *ß-1,3-glucanase*, while callose deposition can be maintained in resistant plants when the expression of *ß-1,3-glucanase* gene is low. However, in contrast to this hypothesis we found that expression of the *BGLU* gene increased during the 24 h of GPA feeding in the leaves of resistant accession PB2013071, but not in leaves of susceptible accession PB2013046. There may be a delicate balance between the expression level of the callose synthesis and callose degrading genes. The *BGLU* accumulation might be caused by the plant’s need to degrade callose in the phloem, as callose deposition may affect the transport of assimilates. The fact that also under the empty clip cages the expression of the *BGLU* gene increased may be related to the involvement of the *BGLU* gene in the general defense response [85–87]. Putting a clip cage on a leaf may inflict such a response.

The accumulation of the *CalS1* and *CalS7* gene transcripts seems not to coincide with impaired phloem uptake as recorded by EPG. The gene expression increased after 6 h infestation whereas aphids already show difficulty in phloem feeding before that time. One possible explanation is that callose deposition is regulated at the protein level in the early stage of the defense response. In bean, callose can be induced within 5–10 min after injury through the activation of proteases [28]. We found that aphids tried to start phloem probing after about 1.5 h on resistant as well as susceptible plants (EPG parameter: time to first E1). However, no callose deposition was detected 1.5 h after the start of the aphid infestation (results not shown), which suggests that callose deposition is not involved in the early response of PB2013071 to aphid feeding. Another possible mechanism of phloem vessel occlusion is plugging by phloem proteins (P-proteins), which can block sieve tubes of higher-level plants rapidly [88–92]. P-proteins based occlusion is thought to be a faster and earlier response than callose deposition [90]. It may be speculated that specific P-proteins are involved in the early response to aphids on the resistant accessions, while callose deposition is induced later to prevent aphid feeding in a more stable and long-lasting way.

## Conclusion

In conclusion, we identified three *C. baccatum* accessions that are resistant to the green peach aphid and one *C. baccatum* accession that is susceptible. Accession PB2013071 shows the highest aphid resistance, which seems to be phloem based according to the EPG recordings. The resistance is accompanied by callose deposition in the sieve elements, which may be at least partially causal. The up-regulated expression of the *CalS1* and *CalS7* genes in the resistant accession is in line with this observation.

## Methods

### Plant materials and growing condition

The plant materials used consisted of accessions of *C. annuum*, *C. chinense*, *C. frutescens* and *C. baccatum* that were obtained from the Centre for Genetic Resources, the Netherlands (CGN) and from the collection of Wageningen University & Research. Based on the results of an initial evaluation of about 50 accessions, additional material from *C. baccatum* were screened. The accession codes, names, and species of all materials used can be found in the Additional file 1: Tables S1 and Additional file 2: Table S2.

Two weeks after sowing, plants were transplanted into 14 cm pots with potting compost and grown in a standard greenhouse at 19–21 °C, 60–70% relative humidity and a 16–8 h light–dark photoperiod at Wageningen University & Research, Wageningen, NL. Plants were watered every other day and no aphid control was applied during growth and testing.

### Aphid population

The GPA (*M. persicae*) population used originated from the population used by [93]. Initially it was reared on Chinese cabbage (*B. rapa*) cv. Granaat; later the rearing was transferred to *C. annuum* accession CGN19226 and subsequently to *C. baccatum* accession PB2013046. The aphid rearing was maintained in a standard greenhouse under the same conditions as the pepper plants.

### Evaluation of *Capsicum* accessions for GPA resistance in a clip cage test

All evaluations were carried out in the greenhouses of Wageningen University & Research, Wageningen, NL and were performed in four experiments during summer and autumn. The first experiment, including 50 accessions (Additional file 1: Table S1), was done when plants were eight weeks old. Plants were tested in a complete block design with four blocks in the same glasshouse compartment, with one plant of each accession per block and two clip cages containing per cage 10 1-day-old GPA nymphs that were obtained from a rearing on Chinese cabbage. The clip cages were placed on the abaxial side of the top two fully expanded leaves of the plants. After seven days the numbers of surviving and dead aphids as well as new nymphs produced in each clip cage were counted. The second experiment was conducted similarly to the first with the following changes. Ten accessions were selected from the 50 tested in the first experiment (Additional file 1: Table S1). They were re-tested in a complete block design with 10 blocks when they were seven weeks old, one plant per accession in each block, again per plant with two clip-on cages with 10 1-day-old nymphs, originating from a rearing on *C. annuum* accession CGN19226.

In the third experiment only *C. baccatum* accessions were evaluated, together with *C. annuum* CGN19226 as susceptible control (Additional file 2: Table S2) in a complete block design with four blocks under conditions similar to the first two experiments. They were evaluated with two clip cages per plant, containing 5 1-day-old GPA nymphs per cage obtained from a rearing on CGN19226, when plants were seven weeks old. During the fourth experiment, eight selected accessions from the third experiment (including the susceptible *C. annuum* CGN19226) were re-tested in a complete block design with five blocks (Additional file 2: Table S2). Similar to the third experiment plants were evaluated with two clip cages containing 5 1-day-old GPA nymphs originated from a rearing on the susceptible *C. baccatum* accession PB2013046, when the plants were seven weeks old. After eight days all clip cages were observed.

For statistical analysis, the observations from two clip cages per plant were combined. Survival was determined by dividing the number of living aphids by the total number of aphids (dead and alive) in the clip cage. The number of new nymphs was divided by the average number of living aphids present, calculated as (2*living aphids + dead aphids)/2. Additionally, data used for ANOVA analysis were transformed to obtain a more or less constant residual variance: survival as arcsin(sqrt(x)) and nymphs as sqrt(x). Significance of differences in the means was evaluated using the LSD test (*P* < 0.05) on the transformed data.

### Population development

A population development experiment was used to further confirm resistance/susceptibility of the accessions. Ten plants of each selected accession were randomized in one greenhouse compartment. Approx. 40 days after sowing each plant was infested with 5 wingless GPA adults and 10 nymphs and enclosed in an aphid-proof sleeve. After 19 days, the number of adult aphids was counted and the number of nymphs was estimated according to a visual scale (0 = none, 1 = few (< 50), 2 = many nymphs (> 50)). For ANOVA analysis, the number of adults per plant was transformed to log(x). Significance of differences of means was tested by LSD test (*P* < 0.05).

### Electrical Penetration Graph

The Electrical Penetration Graph (EPG) technique was used to monitor GPA probing and feeding behaviour on the most resistant (PB2013071) and a susceptible (PB2013046) *C. baccatum* accession. For each accession, 10 seven-week-old plants were each probed with two adult aphids placed on the abaxial side of the top two fully expanded leaves. Experimental setup was as described by [94]. Recording lasted for six hours at 20 ± 2 °C under constant light. The EPG patterns were transformed into waveforms using the Stylet+a software version 1.20 (http://www.epgsystems.eu/). Extraction of resistance parameters from the waveforms was carried out using EPG-Calc 6.1.3 [95]. T-tests were used to determine the significance of the differences between the accessions for various EPG parameters. The Fisher exact test was used to determine the significance of the difference in percentage of aphids that reached E2 during six hours’ recording.

### Callose deposition

Histological analysis of *in situ* callose deposition was performed essentially as described by [96] on the resistant (PB2013071) and susceptible (PB2013046) *C. baccatum* accession. The second fully expanded leaf with petiole was cut with scissors from each plant and immediately put into a 6 cm-diameter petri dish with 1.5% water-agar medium. Twenty randomly selected wingless aphids were put gently into the petri dish, which was sealed by Parafilm M (Bemis NA, USA). Four plants/replicates were used for each treatment or control. After 24 h, three to four leaf disks (1.3 cm in diameter) containing highest number of aphids were sampled from the detached leaf and directly placed in 96% ethanol with their abaxial side up to remove chlorophyll. After washing in 0.07 M K_2_HPO_4_ (pH = 9), leaf disks were stained for 2 h in 0.1% (*w*/*v*) aniline blue in 0.07 M K_2_HPO_4_ (pH = 9) at room temperature. Samples were subsequently mounted on glass slides with 70% glycerol. Callose fluorescence was observed qualitatively under UV light, and photos were taken using the Zeiss Axiophoto digital imaging microscope (Carl Zeiss AG, Germany). Control leaf samples without aphids were treated in the same way; leaf disks were taken from areas comparable to the areas taken from the infested leaves. In total 12 leaf disks were observed for accession PB2013071 and 14 for accession PB2013046 after 24 h GPA treatment; and 12 leaf disks were observed for both accessions as control.

### Gene expression analysis

The expression level of callose related genes was analyzed by quantitative real-time PCR. Seven-week-old plants received three clip cages containing 15 randomly selected wingless aphids per cage. Leaf disks were collected from the clip cage areas 1.5, 6 and 24 h after the start of aphid infestation. After gently brushing aphids away, disks were flash-frozen in liquid nitrogen and stored at − 80 °C until use. Leaf disks under an empty clip cage were also collected after 1.5, 6 and 24 h and used as reference. Additionally, leaf disks without clip cage and aphid infestation were collected just before the infestation stated (time point 0 h). Four biological replicates were used per treatment with aphid infestation and three per treatment with empty clip cages. For the reference without clip cages (time point 0 h) also three biological replicates were used. In all cases, two plants were pooled together as one biological replicate.

The sequences of *CalS* family genes were obtained from the Pepper Genome Platform (http://peppergenome.snu.ac.kr/) [97] and the Pepper Genome Database (http://peppersequence.genomics.cn/page/species/index.jsp) [98] through BlastP queries [99] referring to the sequences from *Arabidopsis* (https://www.arabidopsis.org/index.jsp). Genes were identified and named according to phylogenetic tree of *CalS* family genes among *Arabidopsis*, grapevine and pepper which was constructed by MEGA5 [100]. Besides the *CalS* family genes, the *basic ß-1,3-glucanase* gene (CA03g30020, *BGLU*) was obtained from the Pepper Genome Platform (http://peppergenome.snu.ac.kr/). The pepper *actin* gene (CA12g08730) was used as an internal reference for normalization of gene expression [101]. Gene specific primers were designed using Primer3Plus (www.bioinformatics.nl/cgi-bin/primer3plus/primer3plus.cgi) and are listed in Additional file 6: Table S4.

Total RNA was isolated with the RNeasy plant mini kit (Qiagen, USA) according to the suppliers’ recommendations. After treatment with DNase I (Invitrogen, USA), 1 μg RNA template was reversely transcribed into cDNA using the iScript™ cDNA Synthesis Kit (Bio-Rad, USA). Quantitative real-time PCR was conducted using the iQ™ SYBR Green Supermix (Bio-Rad, USA) and the CFX96 Touch™ Real-Time system (Bio-Rad, USA).

The PCR mix contained 5 μl 2× iQ™ SYBR GREEN Supermix, 0.3 μl forward primer (10 μM), 0.3 μl reverse primer (10 μM) and 2 μl cDNA template with 10-time dilution, into a final volume of 10 μl. Quantitative RT-PCR was performed in duplicate using the following program: 95 °C for 3 min followed by 40 cycles of 95 °C for 15 s, and 60 °C for 1 min. As the primers were designed on the gene sequences from *C. annuum*, the QPCR products were sequenced to validate the region of amplification in *C. baccatum*. Relative expression was calculated with the 2-^ΔΔCt^ method [102]. Independent-samples t-tests on log2-transformed data were used to determine the significance of the differences between certain time points after GPA infestation and no GPA infestation (*P* < 0.05).

## Additional files

Additional file 1: **Table S1.** Evaluation of *Capsicum* accessions for resistance against the aphid *M. persicae*. (DOCX 21 kb)

Additional file 2: **Table S2.** Evaluation of *C. baccatum* accessions for resistance against the aphid *M. persicae*. (DOCX 19 kb)

Additional file 3: **Figure S1.** Histochemical staining of callose in 24 h GPA-infested leaves. Resistant accession PB2013071 (A, C, E, G); susceptible accession PB2013046 (B, D, F, H). Bars = 100 μm. (PDF 275 kb)

Additional file 4: **Table S3.** Callose synthase (*CalS*) genes in *C. annuum*. (DOCX 15 kb)

Additional file 5: **Figure S2.** Phylogenetic analysis of pepper (Ca), *Arabidopsis* (At) and grapevine (Vv) CalS proteins, using the MEGA [100] neighbour-joining algorithm. (PDF 10 kb)

Additional file 6: **Table S4.** Primer sequences used in real-time PCR. (DOCX 14 kb)
