# Supplementary material for: Reduced phloem uptake of Myzus persicae on an aphid resistant pepper accession
Source: BMC Plant Biol. 2018 Jun 27;18:138. doi: 10.1186/s12870-018-1340-3 (PMC6020309; doi:10.1186/s12870-018-1340-3)
Supplement: Supplementary file 1 — Table S1. Evaluation of Capsicum accessions for resistance against the aphid M. persicae. (DOCX 21 kb) [file 12870_2018_1340_MOESM1_ESM.docx]

| **Table S1. Evaluation of *Capsicum* accessions for resistance against the aphid *M. persicae.*** | | | | | | | | | | | | | |
| --- | --- | --- | --- | --- | --- | --- | --- | --- | --- | --- | --- | --- | --- |
| **Accession**  **Nr.** | **Name** | **Species** | **Experiment 1^3^** | | | | **Experiment 2^4^** | | | | |  |  |
|  |  |  | **Survival^1^** | | **Nymphs^2^** | | **Survival** | | **Nymphs** | | | |  |
| CGN23766 | Small Bell Chilly | *C. chinense* | 0.06 | a | 0.00 | a | 0.91 | bc | 0.17 | bc | | |  |
| CGN22829 | Miscucho colorado; PI 152225; 1 SCA no.6 | *C. chinense* | 0.13 | ab | 0.00 | a | 0.81 | b | 0.34 | cd | | |  |
| PB2012022 |  | *C. baccatum* | 0.27 | abcd | 0.01 | ab | 0.55 | a | 0.00 | a | | |  |
| CGN22855 | PI 281428 Selection | *C. chinense* | 0.21 | abc | 0.13 | abcde | 0.91 | bc | 0.53 | de | | |  |
| PB2012024 |  | *C. baccatum* | 0.29 | abcd | 0.00 | a | 0.92 | bc | 0.05 | ab | | |  |
| PB2012036 |  | *C. chinense* | 0.36 | bcdef | 0.00 | a | 0.94 | c | 0.24 | cd | | |  |
| CGN19229 | No. 1882 selection; PI 281417 selection | *C. chinense* | 0.33 | bcde | 0.00 | a | 0.92 | bc | 0.30 | cd | | |  |
| CGN22786 | SA 344; PI 260434 | *C. baccatum* | 0.39 | bcdefg | 0.00 | a |  |  |  | |  |  |  |
| CGN17023 | Aceituno; PI 281443; No.1852 | *C. chinense* | 0.36 | bcdef | 0.01 | ab |  |  |  | |  |  |  |
| CGN23763 | RU 72-77 | *C. baccatum* | 0.51 | cdefgh | 0.01 | ab |  |  |  | |  |  |  |
| CGN16972 | 1 GAA; PI 263258 | *C. baccatum* | 0.52 | cdefghi | 0.00 | a |  |  |  | |  |  |  |
| CGN22775 | 7104 | *C. frutescens* | 0.54 | defghij | 0.00 | a |  |  |  | |  |  |  |
| CGN22096 | I 5429 | *C. baccatum* | 0.51 | cdefgh | 0.00 | a |  |  |  | |  |  |  |
| CGN23278 | PI 337524 | *C. baccatum* | 0.63 | efghijk | 0.00 | a |  |  |  | |  |  |  |
| CGN17042 | No. 1553; PI 238061 | *C. baccatum* | 0.67 | efghijkl | 0.01 | ab |  |  |  | |  |  |  |
| CGN22831 | Pimento | *C. annuum* | 0.64 | efghijk | 0.01 | ab |  |  |  | |  |  |  |
| CGN16975 | AC 1979 | *C. annuum* | 0.69 | fghijkl | 0.00 | a |  |  |  | |  |  |  |
| CGN17221 | Morron Selection; PI 257284 Selection | *C. chinense* | 0.71 | ghijkl | 0.01 | ab |  |  |  | |  |  |  |
| CGN22185 | No.4692; PI 159249; 1SCA | *C. baccatum* | 0.72 | ghijkl | 0.02 | ab |  |  |  | |  |  |  |
| CGN23206 | RU 72-51 | *C. baccatum* | 0.76 | hijklm | 0.00 | a |  |  |  | |  |  |  |
| CGN22181 | Yellow Bouquet | *C. baccatum* | 0.73 | hijkl | 0.01 | ab |  |  |  | |  |  |  |
| CGN17219 | No.4661 Selection; PI 159236 Selection | *C. chinense* | 0.73 | ghijkl | 0.01 | ab |  |  |  | |  |  |  |
| CGN23092 | Local; Krasnyi; VIR 1829 | *C. chinense* | 0.76 | hijklm | 0.04 | ab |  |  |  | |  |  |  |
| CGN22830 | Chili Serrano; PI 281367; No. 999 | *C. annuum* | 0.81 | hijklm | 0.00 | a |  |  |  | |  |  |  |
| CGN22817 | Lombok | *C. frutescens* | 0.79 | hijklm | 0.00 | ab |  |  |  | |  |  |  |
| CGN19199 | AC 1448 | *C. frutescens* | 0.72 | ghijkl | 0.08 | abcd |  |  |  | |  |  |  |
| PB2012025 |  | *C. baccatum* | 0.81 | hijklm | 0.03 | ab |  |  |  | |  |  |  |
| CGN19224 | MI 1/81; Pili Pili | *C. chinense* | 0.78 | hijklm | 0.05 | abc |  |  |  | |  |  |  |
| PB2012018 |  | *C. baccatum* | 0.81 | hijklm | 0.00 | a |  |  |  | |  |  |  |

| **Table S1 (continued)** | | | | | | | | | | | |
| --- | --- | --- | --- | --- | --- | --- | --- | --- | --- | --- | --- |
| **Accession**  **Nr.** | **Name** | **Species** | **Experiment 1** | | | | **Experiment 2** | | | |  |
|  |  |  | **Survival** | | **Nymphs** | | **Survival** | | **Nymphs** | |  |
| CGN17009 | Hungarian Wax 4202-3 | *C. annuum* | 0.77 | hijklm | 0.03 | ab |  |  |  |  |  |
| CGN19194 | Cayenne Long Red Narrow | *C. annuum* | 0.91 | klm | 0.00 | a | 0.92 | bc | 1.24 | f |  |
| CGN22131 | Local | *C. chinense* | 0.82 | hijklm | 0.13 | abcde |  |  |  |  |  |
| CGN19188 | Calcom | *C. annuum* | 0.81 | hijklm | 0.11 | abcd |  |  |  |  |  |
| CGN22790 | AC 1249 | *C. frutescens* | 0.86 | klm | 0.07 | abcd |  |  |  |  |  |
| CGN23211 | SA 218; PI 260459, Malagueta | *C. frutescens* | 0.77 | hijklm | 0.15 | bcde |  |  |  |  |  |
| CGN20503 | Bisbas | *C. annuum* | 0.89 | klm | 0.10 | abcd |  |  |  |  |  |
| CGN16994 | RU 72-194 | *C. chinense* | 0.85 | jklm | 0.18 | bcdef |  |  |  |  |  |
| CGN22862 | No.1720; PI 281426; 1GAA | *C. chinense* | 0.81 | hijklm | 0.18 | bcdef |  |  |  |  |  |
| CGN19189 | California Wonder 300 | *C. annuum* | 0.97 | m | 0.07 | abcd |  |  |  |  |  |
| CGN22173 | Sweet Banana | *C. annuum* | 0.93 | lm | 0.10 | abcd |  |  |  |  |  |
| CGN23210 | SA 137; PI 257155 | *C. frutescens* | 0.74 | hijkl | 0.35 | defg |  |  |  |  |  |
| CGN24363 | Vindunger Mutande | *C. frutescens* | 0.70 | fghijkl | 0.36 | defg |  |  |  |  |  |
| CGN22168 | RU 72-357 | *C. frutescens* | 0.83 | ijklm | 0.34 | cdefg |  |  |  |  |  |
| CGN21554 | Ndungu; Pili Pili | *C. frutescens* | 0.93 | lm | 0.11 | abcd |  |  |  |  |  |
| CGN16995 | RU 72-241 | *C. chinense* | 0.90 | klm | 0.34 | defg |  |  |  |  |  |
| CGN17020 | No.965; PI 281353 | *C. frutescens* | 0.85 | jklm | 0.57 | fg |  |  |  |  |  |
| CGN22792 | SA 252; PI 260478, Aji Chuncho | *C. frutescens* | 0.77 | hijklm | 0.69 | g |  |  |  |  |  |
| CGN22779 | C 307 | *C. frutescens* | 0.88 | klm | 0.80 | g |  |  |  |  |  |
| CGN19226 | Bruinsma Wonder | *C. annuum* | 0.87 | klm | 0.50 | efg | 0.93 | c | 0.47 | de |  |
| PB2012045 |  | *C. frutescens* | 0.93 | lm | 0.56 | fg | 0.93 | c | 0.78 | ef |  |

^1^ Survival refers to fraction of the aphids that survived on an accession after 7 days.

^2^ Nymphs means average number of new nymphs reproduced by every estimated living adult.

^3^ Aphids used in Experiment 1 were reared on Chinese cabbage (*B. rapa*) cv. Granaat.

^4^ Aphids used in Experiment 2 were reared on *C. annuum* accession CGN19226.

Means followed by the same letter within the same column are not significantly different (LSD- test on transformed

scales at P<0.05).
