# Supplementary material for: Reduced phloem uptake of Myzus persicae on an aphid resistant pepper accession
Source: BMC Plant Biol. 2018 Jun 27;18:138. doi: 10.1186/s12870-018-1340-3 (PMC6020309; doi:10.1186/s12870-018-1340-3)
Supplement: Supplementary file 2 — Table S2. Evaluation of C. baccatum accessions for resistance against the aphid M. persicae. (DOCX 19 kb) [file 12870_2018_1340_MOESM2_ESM.docx]

| **Table S2. Evaluation of *C. baccatum* accessions for resistance against the aphid *M. persicae.*** | | | | | | | | | | | |
| --- | --- | --- | --- | --- | --- | --- | --- | --- | --- | --- | --- |
| **Accession**  **Number** | **Name** | **Experiment 3^3^** | | | | **Experiment 4^4^** | | | | |  |
|  |  | **Survival^1^** | | **Nymphs^2^** | | **Survival** | | **Nymphs** | | |  |
| PB2013071 |  | 0.51 | ab | 0.000 | a | 0.37 | a | 0.00 | a | |  |
| PB2013062 |  | 0.57 | abc | 0.000 | a | 0.82 | cd | 0.02 | ab | |  |
| CGN23260 | PI 260567 | 0.63 | abcde | 0.000 | a | 0.75 | bc | 0.21 | c | |  |
| CGN22834 | RU 72-48 | 0.73 | abcdefg | 0.000 | a | 0.98 | e | 0.16 | bc | |  |
| PB2013074 |  | 0.76 | abcdefgh | 0.000 | a |  |  |  |  | |  |
| CGN16972 | 1 GAA; PI 263258 | 0.77 | abcdefgh | 0.000 | a |  |  |  |  | |  |
| PB2012022 |  | 0.82 | bcdefghij | 0.000 | a | 0.50 | ab | 0.02 | ab | |  |
| PB2012024 |  | 0.83 | bcdefghij | 0.000 | a |  |  |  | |  |  |
| CGN22096 | I 5429 | 0.85 | cdefghij | 0.000 | a |  |  |  | |  |  |
| CGN21513 | PI 260580 | 0.89 | efghij | 0.000 | a |  |  |  | |  |  |
| CGN23763 | RU 72-77 | 0.94 | ghij | 0.000 | a |  |  |  | |  |  |
| PB2012018 |  | 0.92 | fghij | 0.006 | ab |  |  |  | |  |  |
| CGN22858 | RU 72-93 | 0.59 | abcd | 0.018 | abc | 0.86 | cde | 0.16 | | bc |  |
| CGN21514 |  | 0.85 | cdefghij | 0.018 | abc |  |  |  | |  |  |
| CGN17025 | No. 1553; PI 281306 | 0.94 | ghij | 0.019 | abc |  |  |  | |  |  |
| PB2013061 |  | 0.52 | ab | 0.020 | abc |  |  |  | |  |  |
| CGN22786 | SA 344; PI 260434 | 0.66 | abcdef | 0.025 | abc |  |  |  | |  |  |
| CGN21582 | AC 2129 | 0.85 | cdefghij | 0.029 | abc |  |  |  | |  |  |
| CGN17042 | No. 1553; PI 238061 | 0.97 | hij | 0.029 | abc |  |  |  | |  |  |
| CGN22872 | PM 593 | 0.90 | efghij | 0.030 | abc |  |  |  | |  |  |
| CGN17174 | Aji; VIR 252 | 0.94 | ghij | 0.051 | abcd |  |  |  | |  |  |
| CGN23566 | AC 2060 | 0.64 | abcdef | 0.058 | abcd |  |  |  | |  |  |
| CGN17241 | AC 2200 | 0.98 | ij | 0.090 | abcd |  |  |  | |  |  |
| CGN21512 | PI 260561 | 0.93 | ghij | 0.092 | abcd |  |  |  | |  |  |
| CGN23278 | PI 337524 | 0.77 | abcdefgh | 0.098 | abcd |  |  |  | |  |  |
| CGN21479 | AC 1986 | 0.88 | defghij | 0.099 | abcd |  |  |  | |  |  |
| CGN22871 | PM 325 | 0.93 | ghij | 0.101 | abcd |  |  |  | |  |  |
| CGN22185 | No.4692; PI 159249; 1SCA | 0.98 | j | 0.112 | abcd |  |  |  | |  |  |
| CGN23206 | RU 72-51 | 0.89 | efghij | 0.128 | abcd |  |  |  | |  |  |
| CGN22181 | Yellow Bouquet | 0.97 | hij | 0.128 | abcd |  |  |  | |  |  |
| PB2012025 |  | 0.90 | efghij | 0.179 | bcd |  |  |  | |  |  |
| CGN21515 |  | 0.49 | a | 0.229 | cd |  |  |  | |  |  |
| CGN19233 | Pen 3.4 | 0.89 | efghij | 0.265 | cd |  |  |  | |  |  |
| CGN19202 | Cluster Rod | 0.93 | ghij | 0.269 | cd |  |  |  | |  |  |
| CGN16905 |  | 0.96 | ghij | 0.324 | de |  |  |  | |  |  |
| CGN19226 | Bruinsma Wonder (*C. annuum*) | 0.98 | hij | 0.342 | de | 0.95 | de | 2.32 | | d |  |
| CGN16973 | SA 361 | 0.79 | abcdefghi | 0.370 | de |  |  |  | |  |  |
| PB2013046 |  | 0.94 | ghij | 0.890 | e | 0.98 | e | 1.91 | | d |  |

^1^ Survival refers to fraction of the aphids that survived on an accession after 7 days in Experiment 3 and after 8 days in Experiment 4.

^2^ Nymphs means average number of new nymphs reproduced by every estimated living adult.

^3^ Aphids used in Experiment 3 were reared on *C. annuum* accession CGN19226.

^4^ Aphids used in Experiment 4 were reared on *C. baccatum* accession PB2013046.

Means followed by the same letter within the same column are not significantly different (LSD- test on transformed scales at P<0.05).
