# Supplementary figures and images for: Reduced phloem uptake of Myzus persicae on an aphid resistant pepper accession
Source: BMC Plant Biol. 2018 Jun 27;18:138. doi: 10.1186/s12870-018-1340-3 (PMC6020309; doi:10.1186/s12870-018-1340-3)

**2013071**

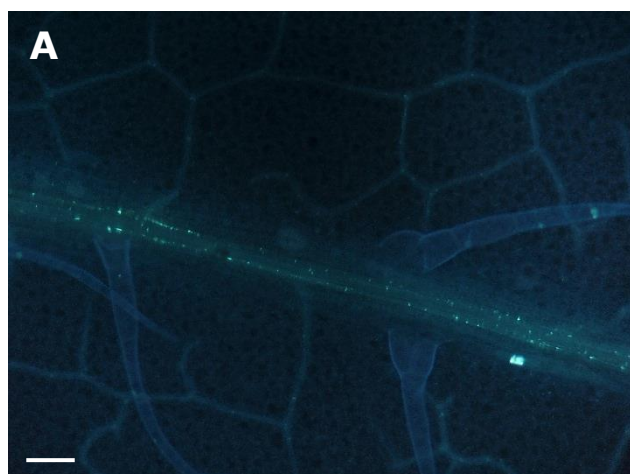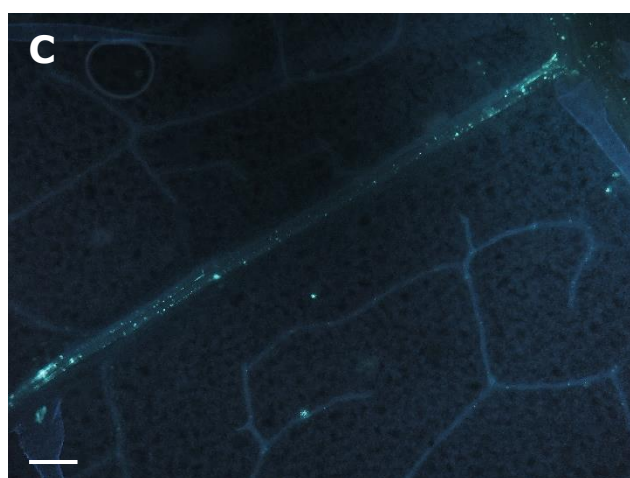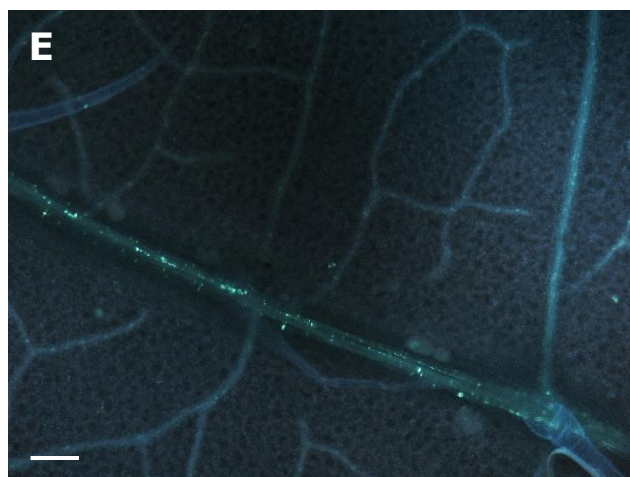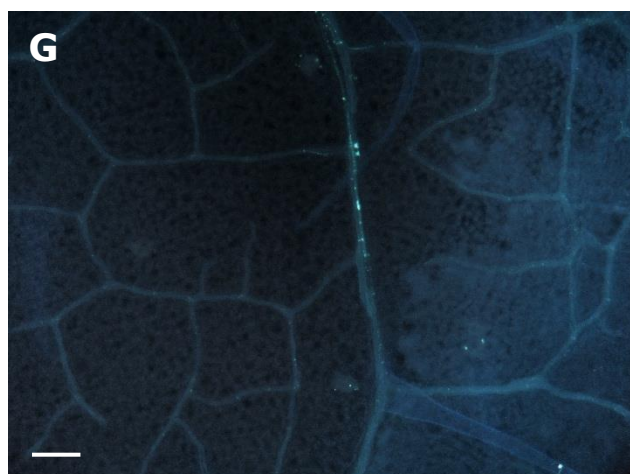

**2013046**

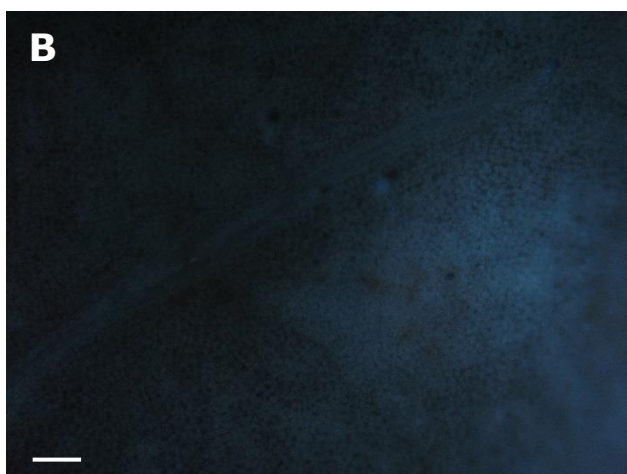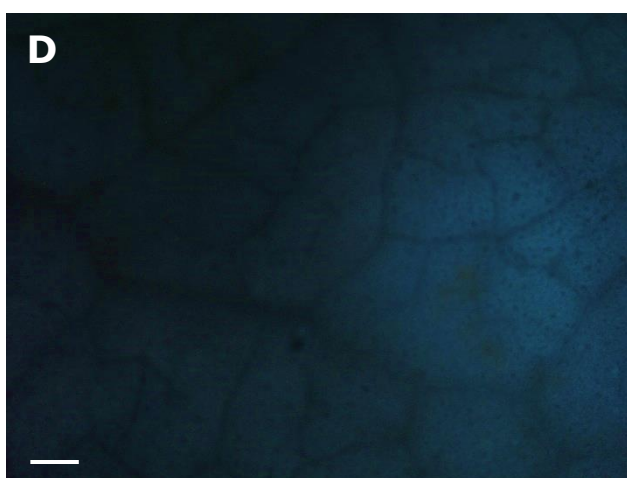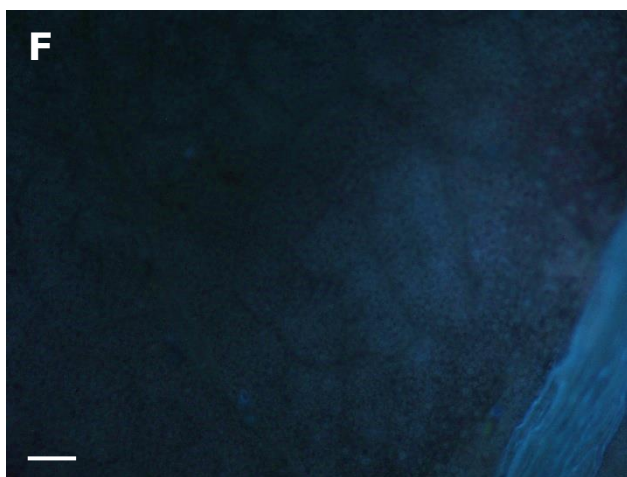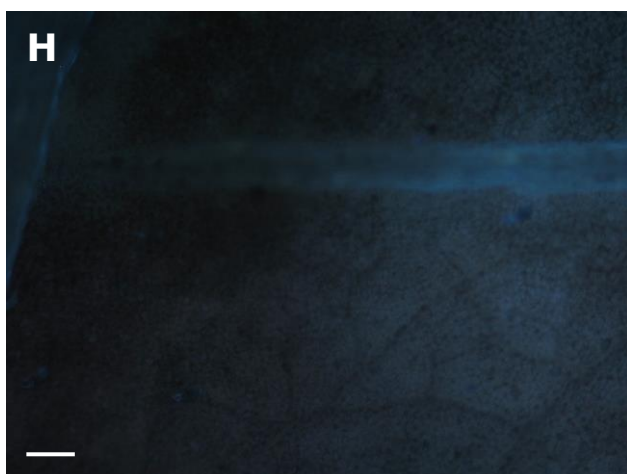

Supplement: Supplementary file 3 — Figure S1. Histochemical staining of callose in 24 h GPA-infested leaves. Resistant accession PB2013071 (A, C, E, G); susceptible accession PB2013046 (B, D, F, H). Bars = 100 μm. (PDF 275 kb) [file 12870_2018_1340_MOESM3_ESM.pdf]

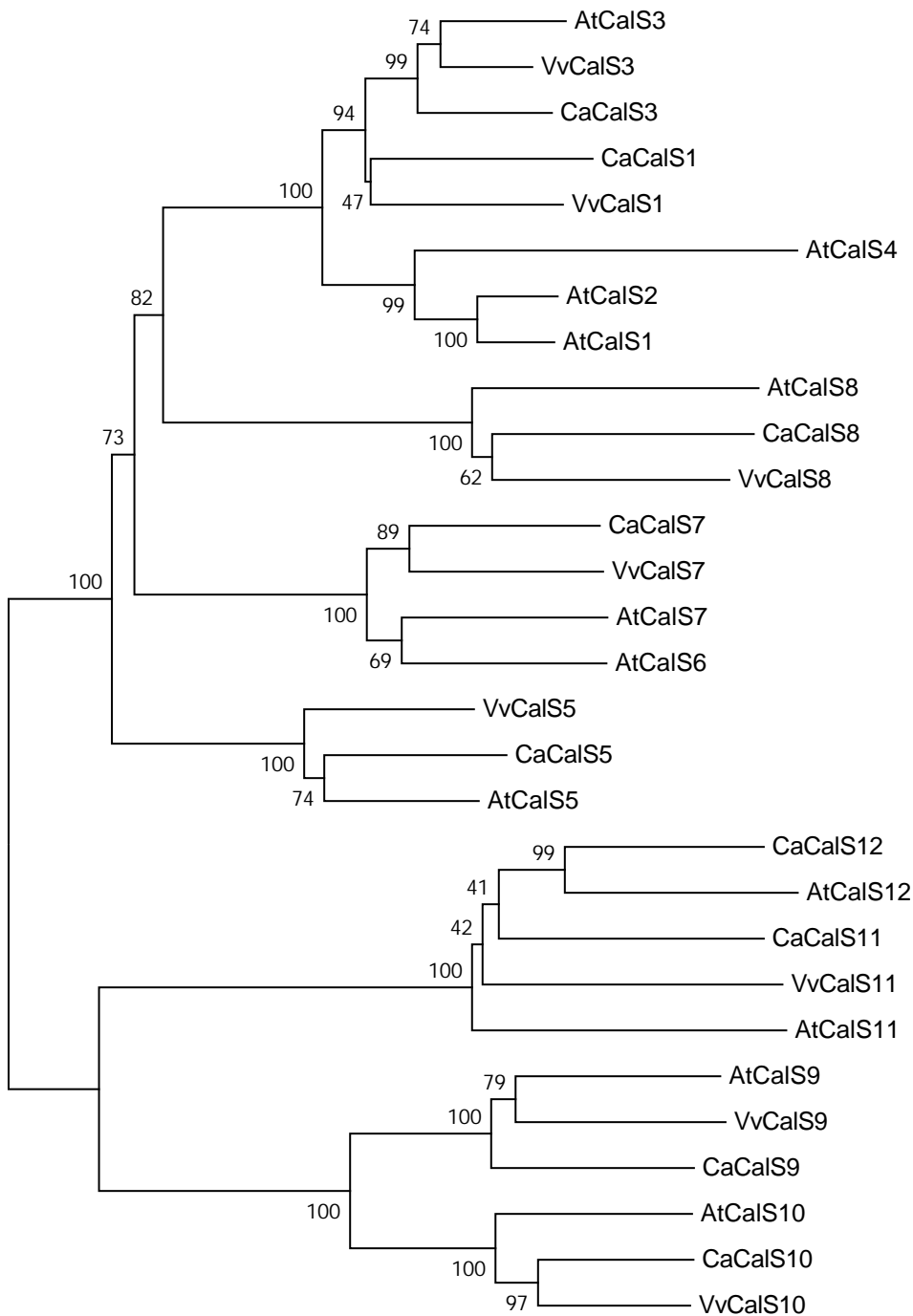

0.05

Supplement: Supplementary file 5 — Figure S2. Phylogenetic analysis of pepper (Ca), Arabidopsis (At) and grapevine (Vv) CalS proteins, using the MEGA [100] neighbour-joining algorithm. (PDF 10 kb) [file 12870_2018_1340_MOESM5_ESM.pdf]
