# Supplementary material for: Reduced phloem uptake of Myzus persicae on an aphid resistant pepper accession
Source: BMC Plant Biol. 2018 Jun 27;18:138. doi: 10.1186/s12870-018-1340-3 (PMC6020309; doi:10.1186/s12870-018-1340-3)
Supplement: Supplementary file 4 — Table S3. Callose synthase (CalS) genes in C. annuum. (DOCX 15 kb) [file 12870_2018_1340_MOESM4_ESM.docx]

**Table S3. Callose synthase (*CalS*) genes in *C. annuum*.**

| **Gene^a^** | **ORF(bp)** | **Gene ID in CM334^b^** | **Gene ID in Zunla1^c^** |
| --- | --- | --- | --- |
| *CaCalS1* | 5856 | CA01g26370 | Capana01g003121 |
| *CaCalS3* | 5847 | CA01g11310 | Capana01g001537-1540 |
| *CaCalS5* | 5460 | CA12g07860-07870 | Capana12g002111 |
| *CaCalS7* | 5730 | CA07g15510 | Capana07g001971 |
| *CaCalS8* | 5865 | CA07g16450 | Capana07g002034 |
| *CaCalS9* | 6393 | CA01g11290-11300 | Capana01g001540 |
| *CaCalS10* | 5265 | CA03g25210 | Capana03g001312 |
| *CaCalS11* | 5316 | CA02g16100 | Capana02g001499 |
| *CaCalS12* | 5307 | CA07g13560 | Capana07g001693 |

^a^ The name of each *C. annuum CalS* (*CaCalS*) gene was assigned on the basis of homology with the *A. thaliana CalS* (*AtCalS*) and *V. vinifera* *CalS* (*VvCalS*) genes*.*

^b^ Pepper Genome Platform (<http://peppergenome.snu.ac.kr/>)

^c^ Pepper Genome Database (<http://peppersequence.genomics.cn/page/species/index.jsp>)
