# Supplementary material for: Reduced phloem uptake of Myzus persicae on an aphid resistant pepper accession
Source: BMC Plant Biol. 2018 Jun 27;18:138. doi: 10.1186/s12870-018-1340-3 (PMC6020309; doi:10.1186/s12870-018-1340-3)
Supplement: Supplementary file 6 — Table S4. Primer sequences used in real-time PCR. (DOCX 14 kb) [file 12870_2018_1340_MOESM6_ESM.docx]

**Table S4. Primer sequences used in real-time PCR.**

| **Gene Name** | **Forward sequence (5’-3’)** | **Reverse sequence (5’-3’)** |
| --- | --- | --- |
| *CalS1* | GCAACCCAAGGGTAGCTTATC | AACTCTGCATTTCACGAGCA |
| *CalS3* | CGTGTCGCGTATCTATGTCG | CGTGTCGCGTATCTATGTCG |
| *CalS5* | ACGTTTAGAGCGTGACAATG | TTCAAAAAGCACTCCAGCAG |
| *CalS7* | ATCCTGCTGCTGGTGAAGAT | ATGTCTTGAATTGACGAACGCC |
| *CalS8* | TCGAGTGGCTTATCTTTGTCG | CAGTCCTTTCCTTCCTTTTCC |
| *CalS9* | CGAGGACCCTAATGTCTCCA | TCTTGGCTTCTATCAATCGTC |
| *CalS10* | TTACGGAGGAGTTGAGAAGGA | GGGTAGCTGAGGGAACTGCT |
| *CalS11* | TGGCTTGGACTCTTCTTTGG | AGCGAACTTGCGACTTCTTC |
| *CalS12* | CGGTAGATGAAGAACCATACAACA | GCCAGTCAAGCAGGTCATAAT |
| *BGLU* | CATTGATATAGCAGGGGGTCA | CAATGTTGGAGCCTCTTAAAGC |
| *Actin* | TGAGCAGGAGCTTGAAACTG | CTTGTCCATCAGGCAATTCA |
